# The Good Old Times

**Published:** 1884-06

**Authors:** 


### THE GOOD OLD TIMES.

THE more you find out about the much vaunted "good old times" the better pleased you are not to have lived in them. The people did not only live like dogs, but hey fed like hogs. A pauper in a workhouse would kick now at a meal which a noble used to devour then. The roast beef of Old England was unheard of; beef was only eaten salted and boiled, and bread was a great luxury, not in common use even by the nobles. The recorder of the Percy family, in the time of Henry VII, shows the extreme coarseness of the mode of living, and an extract or two from the household book of that famous family would give a good idea of the manner in which the most famous noble of the time lived. The permanent household numbered 166 persons and the average of guests was fifty, and the whole of the washing for these 216 persons was for one year 40s., a sum probably equal to $2 in the present day, most of which was for the chapel linen.

From Midsummer to Michaelmas was the only time they indulged in fresh meat, and the instructions say, "My Lord has on his table for breakfast, at 7 in the morning, a quart of beer and wine, two pieces of salt fish, six red herrings, four white ones, and on flesh days, half a chine of beef or mutton boiled." At dinner, men ranking as Knights had a table-cloth, which was washed once a month; and as they had no napkins, and the fingers were extensively used in feeding, this portion at least of their linen must have been in a sad condition. Until the thirteenth century, straw was the bed of Kings; and before that date the King and his family slept in the chamber. The first change was to throw a coverlid over the sleeper; then another was used, and the persons undressed, their linen being substituted for blankets. Beatrice says she would as lief "sleep in a woolen," which shows that such a thing was done even in Shakespeare's time. The use of nothing but coarse dirty woolen next the skin, seldom changed, and the heavy, exciting nature of highly-salted food, on which all lived, of course tended to produce those diseases for which hospitals were founded all over England, hospitals for leprosy in particular abounding.

